# Defined Antigen Skin Test for Bovine Tuberculosis Retains Specificity on Revaccination With Bacillus Calmette–Guérin

**DOI:** 10.3389/fvets.2022.814227

**Published:** 2022-04-13

**Authors:** Saraswathi Subramanian, Sreenidhi Srinivasan, Kathiravan Ramaiyan Selvaraju, Priyadharshini Michael Vinoli, Suganya Selladurai, Boominathan Ramasamy, Karthik Kumaragurubaran, Douwe Bakker, Martin Vordermeier, Vivek Kapur, Dhinakar Raj Gopal

**Affiliations:** ^1^Translational Research Platform for Veterinary Biologicals, Centre for Animal Health Studies, Tamil Nadu Veterinary and Animal Sciences University, Chennai, India; ^2^The Huck Institutes of the Life Sciences, The Pennsylvania State University, University Park, PA, United States; ^3^Central University Laboratory, Tamil Nadu Veterinary and Animal Sciences University, Chennai, India; ^4^Technical Consultant and Independent Researcher, Lelystad, Netherlands; ^5^Department of Bacteriology, Animal and Plant Health Agency, Addlestone, United Kingdom; ^6^Centre for Bovine Tuberculosis, Institute for Biological, Environmental and Rural Sciences, University of Aberystwyth, Aberystwyth, United Kingdom; ^7^Department of Animal Science, The Pennsylvania State University, University Park, PA, United States; ^8^Department of Animal Biotechnology, Madras Veterinary College, Tamil Nadu Veterinary and Animal Sciences University, Chennai, India

**Keywords:** bovine tuberculosis (bTB), Bacillus Calmette-Guérin (BCG) vaccine, DST, specificity, DIVA

## Abstract

The Bacillus Calmette–Guérin (BCG) vaccination provides partial protection against, and reduces severity of pathological lesions associated with bovine tuberculosis (bTB) in cattle. Accumulating evidence also suggests that revaccination with BCG may be needed to enhance the duration of immune protection. Since BCG vaccine cross-reacts with traditional tuberculin-based diagnostic tests, a peptide-based defined antigen skin test (DST) comprising of ESAT-6, CFP-10, and Rv3615c to detect the infected among the BCG-vaccinated animals (DIVA) was recently described. The DST reliably identifies bTB-infected animals in experimental challenge models and in natural infection settings, and differentiated these from animals immunized with a single dose of BCG in both skin tests and interferon-gamma release assay (IGRA). The current investigation sought to assess the diagnostic specificity of DST in calves (*Bos taurus* ssp. *taurus* × *B. t*. ssp. *indicus*; *n* = 15) revaccinated with BCG 6 months after primary immunization. The results show that none of the 15 BCG-revaccinated calves exhibited a delayed hypersensitivity response when skin tested with DST 61 days post-revaccination, suggesting 100% diagnostic specificity (one-tailed lower 95% CI: 82). In contrast, 8 of 15 (diagnostic specificity = 47%; 95% CI: 21, 73) BCG-revaccinated calves were positive per the single cervical tuberculin (SCT) test using bovine tuberculin. Together, these results show that the DST retains its specificity even after revaccination with BCG and confirms the potential for implementation of BCG-based interventions in settings where test-and-slaughter are not economically or culturally feasible.

## Introduction

Bovine tuberculosis (bTB) is a chronic infectious disease that is caused by members of the *Mycobacterium tuberculosis* complex (MTBC) in cattle (1). The disease is a significant threat to public health and continues to be endemic in most low- and middle-income countries (LMICs) contributing to an estimated annual economic burden of $3 billion globally (2). Bovine TB is well-controlled in most high-income countries due to the implementation of test-and-cull strategies. For example, a test-and-cull-based control program was implemented in 1917 in the United States, and together with robust surveillance strategies and pasteurization of milk, this program has been successful in effectively controlling bTB. As part of this program, ~230 million cattle were screened, and ~3.8 million were culled (3, 4). However, similar strategies at this scale are not feasible in LMICs due to both social and economic reasons. Hence, there is an urgent need for alternate solutions such as vaccination that can help control the disease in these settings (5).

The Bacillus Calmette–Guérin (BCG) vaccine was first experimentally used in cattle in 1912, much before its use in humans (6). BCG vaccine-induced protection, although limited, and reduction in pathology in cattle have been repeatedly demonstrated (7–9), but it was not pursued for field use mainly because of its interference with the current World Organization for Animal Health (OIE)-recommended tuberculin-based skin tests. Due to the presence of cross-reactive antigens, BCG compromises the diagnostic specificity of the tuberculin skin test, rendering it incompatible for use in tuberculin-based intervention programs (10). However, per recent data, BCG may have an important role to play in endemic LMIC settings, and therefore, its efficacy against bTB in the field needs to be rigorously evaluated (5). A vast majority of the studies conducted thus far, were performed in experimental settings that are only capable of measuring a reduction in susceptibility (direct vaccine effect) and not its impact on transmission (indirect effect) (11, 12). A recent study performed quantitative meta-analysis and constructed scenario plots taking into account modest direct (from meta-analysis) and indirect (from published natural transmission studies) effects and showed the possibility of gaining considerable success in terms of cumulative cases averted upon immediate implementation of BCG in high burden settings (13). These analyses suggest that BCG vaccination may help accelerate control of bTB in endemic settings, particularly with early implementation in the face of dairy intensification in regions that currently lack effective bTB control programs (14).

In the case of implementation of a BCG-based intervention strategy, there is an urgent need for the development and validation of a diagnostic test that can detect the infected among vaccinated animals (DIVA) (10, 15, 16). Extensive research has been performed to identify antigens with DIVA capability through comparative genomic and transcriptomic analyses, and ESAT-6, CFP-10, and Rv3615c have been identified as the most promising antigens thus far. These antigens are present in field strains of *M. bovis* and other pathogenic members of the MTBC, but are either absent or not immunogenic in the widely used vaccine strain, BCG (10, 17, 18). A novel peptide-based defined antigen skin test (DST) comprising these three antigens was developed, and its sensitivity in reliably identifying infected animals was assessed in both experimental and field settings (19). Further, we recently assessed the diagnostic specificity of DST in skin tests in BCG vaccinates (*Bos taurus* ssp. *taurus* x *B. t*. ssp. *indicus*) under field conditions to be 100% (one-tailed lower 95% CI: 82), whereas the PPD-based single cervical tuberculin (SCT) test had a specificity of 33% (95% CI: 12, 62). However, given the fact that the protection imparted following a single dose of BCG is not complete, and revaccination is required to boost protection against bTB, a follow-up study was performed wherein the vaccinates in the previous study were revaccinated (*n* = 15) after a 6-month interval, and control calves received their first dose of BCG (*n* = 14). Here, the specificity of DST in revaccinates is assessed and compared with that of vaccinates (single dose).

## Materials and Methods

### Animals and Experimental Design

**Figure 1:**
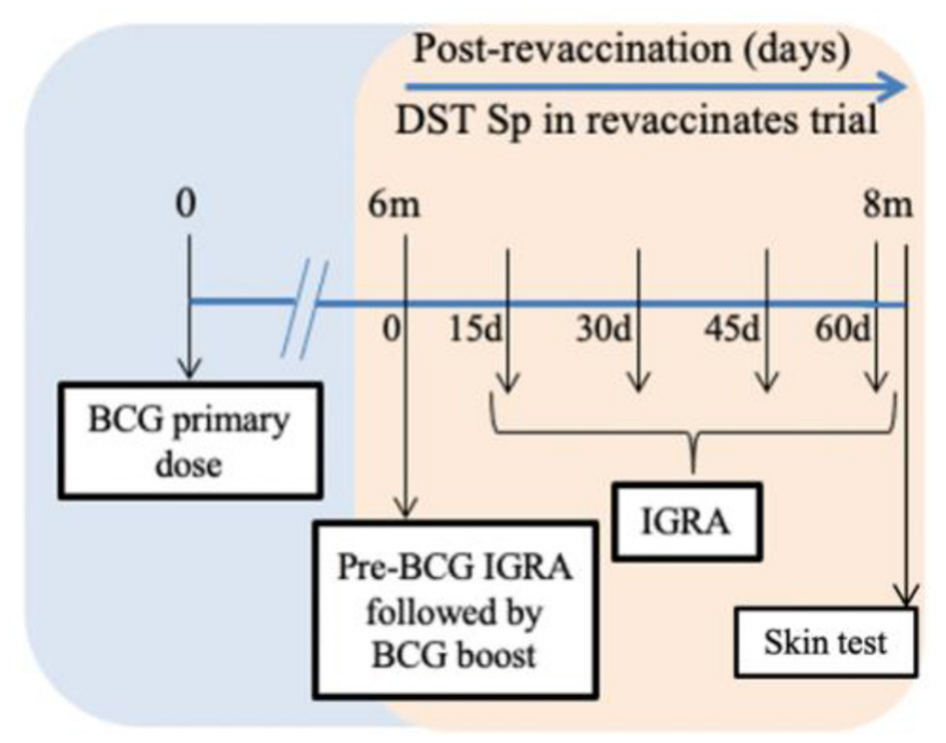
Timeline chart. Calves received the first Bacillus Calmette–Guérin (BCG) dose on day 0 of the previous trial (blue section) (20). The orange section represents the current trial. BCG revaccination was performed after 6 months of the first dose (day 0 of current trial). Blood was collected for interferon-gamma release assay (IGRA) just prior to revaccination with BCG and on days 15, 30, 45, and 60 post revaccination. Skin test was conducted on day 61 post-revaccination. The duration of the current trial from revaccination to skin test is 2 months.

The cattle experiments were conducted under field conditions (normal animal husbandry practices) at the Tamil Nadu University of Veterinary and Animal Sciences (TANUVAS), Chennai, India, and approved by the Institutional Animal Ethics Committee (IAEC) and Committee for the Purpose of Control and Supervision of Experimental Animals (CPCSEA; F. No. 25/31/2017-CPCSEA). Following the initial recruitment of calves in May 2019, the animals were screened for helminths and dewormed during the acclimatization period of 2 weeks. In the previously published study, there were two groups (BCG vaccinates and controls) of 15 calves (*B. taurus* ssp. *taurus* × *B. taurus* ssp. *indicus*) each, wherein the specificity of DST following a single dose of BCG was established. Here, in continuation of the previous study (20), the group of crossbred calves that was previously vaccinated with BCG, received the second BCG dose after a 6-month interval (*n* = 15). The control group from the previous study received the first dose of BCG at the same time (*n* = 14; one calf died during the current trial). Here, we compared the performance of DST in revaccinates with that of vaccinates. The calves were 9–12 months old at the time of vaccination in this study. The calves were fed with dry and green fodder along with concentrate feed containing a balanced mix of grains, brans, minerals, vitamins, and water *ad libitum* during the trial period. The trial timeline is shown in Figure 1.

### Bacillus Calmette–Guérin Vaccination

The lyophilized human vaccine *M. bovis* BCG Danish 1331 was used to vaccinate the animals. Freeze-dried BCG vials were obtained from Green Signal Bio Pharma Pvt. Ltd. The vaccine was prepared as per manufacture's instructions by reconstituting each freeze-dried BCG vial with 1 ml of 0.9% NaCl and the number of colony-forming units (CFUs) was determined by plating 10-fold dilutions on modified 7H11 agar plates (21). The plates were sealed with parafilm and incubated at 37°C for 28 days. A single dose of 0.5 ml of this suspension (1–4 × 10^6^ CFU) was administered subcutaneously to both the groups (vaccinates and revaccinates). The BCG dose was decided based on previous studies, which have shown relatively low doses of 10^4^-10^6^ CFU administered subcutaneously to be efficacious. This dose is the equivalent of five human doses and has been shown in numerous studies to impart a significant degree of protection based on the reduced proportion of vaccinated cattle to develop bTB, as well as presenting with reduced bTB severity (8, 10). However, there is lack of reliable data available on BCG revaccination intervals. Here, for the objective of determining specificity of DST post-revaccination, we used a stringent 6-month revaccination interval.

### Antigens

A total of 13 peptides covering the sequences of ESAT-6, CFP-10, and Rv3615c were commercially synthesized at >98% purity by GenScript USA, Inc. and USV Private Limited, India (Supplementary Table S1 for peptide sequences). The identity was confirmed by mass spectrometry. A cocktail of these 13 peptides was prepared and is henceforth referred to as the defined antigen skin test (DST). A safety trial for DST at escalating and repeated doses was conducted in *Bos taurus* ssp. *indicus* crossbred cattle under good laboratory practice (GLP) conditions in India (data not shown). Avian and bovine tuberculin (PPD-A and PPD-B; purchased from Prionics, Thermo Fisher, Schlieren, Switzerland) are at target concentrations of 0.5 and 1 mg/ml with a potency of 25,000 and 30,000 IU/ml, respectively.

### Skin Test Procedures

A cocktail of the 13 peptides constituting the DST was prepared at 10 μg per peptide. One dose of DST comprises a total peptide quantity of 130 μg. This was injected in a 0.1 ml volume solution. The DST dose is based on prior dose titration experiments performed in crossbred calves (*B. taurus* × *B. taurus* ssp. *indicus*) (15). The PPD–tuberculins (stock PPD-B at 30,000 IU/ml and PPD-A at 25,000 IU/ml) were injected in a 0.1-ml volume as recommended by the manufacturer and the OIE. The skin test was performed in accordance with OIE guidelines (22). McLintock syringe (Bar Knight McLintock Limited, UK) was used, and the injection sites were cleaned with 70% ethanol and shaved prior to injection. Proper administration of the antigen was confirmed by palpating a small pea-like swelling at each site of injection. Injection sites for the three antigens (PPD-A, PPD-B, and DST) were randomized using a Latin square design to account for any site-specific effects. The skin thickness was measured by the same operator prior to and 72 h post-injection. Results are expressed as the difference in skin thicknesses (mm) between the pre- and post-skin test readings. The skin test was performed 2 months post-revaccination (day 61), which was 4 months after the last skin test (conducted at the end of the previous study). For DST, the 2 mm cut-off had been defined previously by performing receiver operating characteristic (ROC) curve analysis. However, we would like to acknowledge that this cutoff will be constantly under review, particularly using large-scale field trial data when available, to ensure that they provide the optimal balance between sensitivity and specificity.

Blood was collected for IGRA just prior to BCG revaccination on day 0 and on days 15, 30, 45, and 60 post-vaccinations from both groups. Whole blood was collected in heparin tubes, and blood cultures were stimulated with PPD-B and PPD-A at final concentrations of 300 and 250 IU/ml, respectively (antigens provided with the BOVIGAM^TM^ kit were not used), on the same day. The DST cocktail (13 peptides) was used at a final concentration of 1 μg/ml for stimulation. This concentration of DST was decided based on systematic dose titration experiments conducted with known reactors and non-reactors in the field (data not shown). Cultures were set up within 8 h of blood collection and whole blood was stimulated at 37°C in 5% CO_2_ overnight. Plasma culture supernatants were separated and stored at −80°C (due to BOVIGAM kit shortage). The ELISA for both groups from a particular time point was later conducted on the same day to determine IFN-γ concentrations using the BOVIGAM^TM^ kit (Thermo Fisher Scientific, USA). Nil-antigen controls were included for every sample, and results for antigen-stimulated cultures are expressed as background-corrected optical density at 450 nm (Δ*OD*_450_). Data interpretation was based on the BOVIGAM kit instruction manual. A reaction was considered positive if the optical density (OD) value of bovine PPD subtracted from avian PPD was greater than or equal to 0.1.

### Statistical Analysis

All statistical analyses were performed using Prism 9 (GraphPad Software, Inc., La Jolla, CA, USA). The IGRAs were performed at five timepoints. One-way ANOVA and Tukey's multiple comparison test was used to determine statistical significance between the various timepoints in IGRA, and various antigens in skin test.

## Results

### DST Elicits a Highly Specific INF-γ Response in Calves Revaccinated With BCG

**Figure 2:**
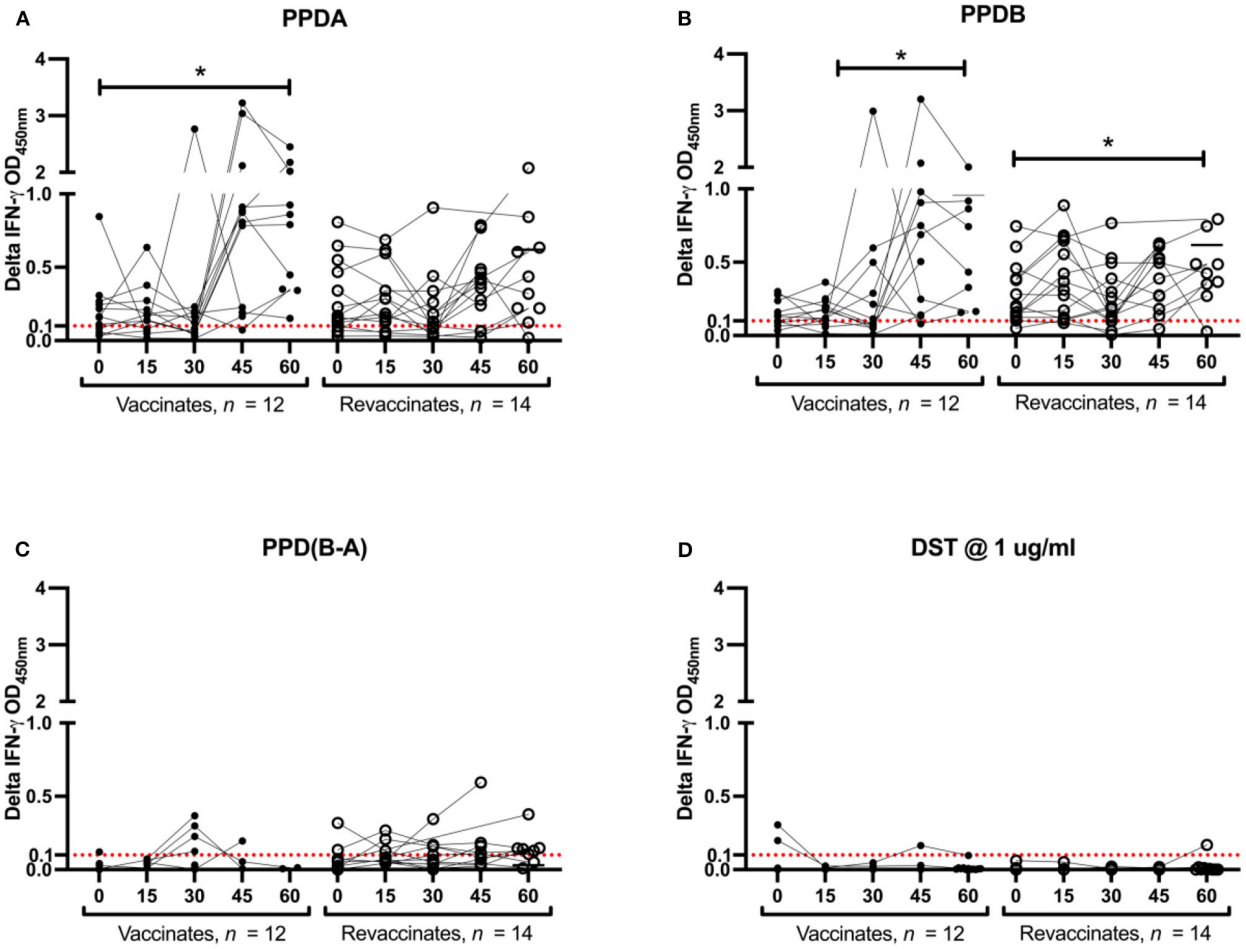
PPDs- and DST-stimulated IFN-γ responses following Bacillus Calmette–Guérin (BCG) vaccination. *In vitro* IFN-γ responses of BCG vaccinates (closed circle, *n* = 12) and revaccinates (open circle, *n* = 14) to **(A)** PPD-A, **(B)** PPD-B, **(C)** PPD(B-A), and **(D)** DST. Results are expressed as background-corrected (delta) optical density (OD) values. Days post-BCG are shown in the x-axis. All calves were vaccinated with BCG on day 0. Blood was collected for interferon-gamma release assay (IGRA) just prior to injection of BCG on day 0, and on days 15, 30, 45, and 60 post-vaccination. The differences in responses induced between the various timepoints were determined using Tukey's multiple comparison test (*p < 0.05). The IGRA cutoff of 0.1 is shown as a dotted red line.

In order to evaluate the DIVA capability of DST *in vitro* in vaccinates (*n* = 14) and revaccinates (*n* = 15), whole-blood was collected on days 0, 15, 30, 45, and 60 post-BCG from all animals. Note that IGRA data are only available for 12 vaccinates and 14 revaccinates as 2/14 vaccinates and 1/15 revaccinate had to be eliminated due to unstimulated (media) control sample exceeding the threshold of 0.3 OD at 450 nm. These data could not be repeated due to BOVIGAM shortage and COVID-related logistic issues. Alongside DST, the PPDs procured from Thermo Scientific were also used for *in vitro* stimulation of whole-blood cultures. Blood was collected just before administration of BCG on day 0 of the current trial, and it was observed that both revaccinates (those that had been vaccinated 6 months before) and vaccinates (first BCG dose on day 0 of current trial) were eliciting IFN-γ responses to the PPDs. This non-specific response to PPDs is likely due to cross-reaction with antigens from environmental mycobacteria, the burden of which is high in settings like India (23, 24). It is important to note that all animals were housed together through the entire duration of the trial. The non-specific reactions to PPDs in the revaccinate group may also be attributed to the residual cross-reactivity with the first dose of BCG. At day 0 (6 months since the first dose of BCG) of the current trial, PPD-A and PPD-B elicited a mean INF-γ response of 0.21 ± 0.06 and 0.25 ± 0.10 in vaccinates, and 0.28 ± 0.07 and 0.28 ± 0.06 in revaccinates, respectively. While considering the IGRA (B-A) cutoff of 0.1, 2/12 vaccinates and 2/14 revaccinates were identified as reactors on day 0. The PPD-stimulated IFN-γ responses increased significantly from the 0-day baseline levels at day 60 in both vaccinates (PPD-A: 1.14 ± 0.23; PPD-B: 0.96 ± 0.18) and revaccinates (PPD-A: 0.80 ± 0.17; PPD-B: 0.78 ± 0.14) (Figures 2A,B; Table 1). At the last time point (day 60), the mean responses induced by the PPDs in vaccinates were higher than those in revaccinates. While 2/12 vaccinates were also identified as reactors per DST in IGRA at day 0, there was no significant increase in the elicited response from day 0, and only a minimal mean response (0.01 ± 0.01) was observed in both vaccinates and revaccinates on day 60 (Figures 2C,D; Table 1). We note here that most animals at this timepoint were still young calves, exhibiting increased false-positive rates due to natural killer (NK) cell activity (25). At the final timepoint for IGRA (day 60 post BCG), 6/14 and 1/14 revaccinates were identified as reactors per IGRA (B-A) and DST, respectively. The total number of reactors, observed mean and standard errors, and across all timepoints for the antigens used are summarized in Table 1.

**Table 1:** The total number of animals that crossed the interferon-gamma release assay (IGRA) cutoff of 0.1 for each antigen tested; mean IGRA responses stimulated by each antigen tested with standard error of the mean is shown in parenthesis.

**Group**	**Antigen**	**Days**				
		**0**	**15**	**30**	**45**	**60**
**Revaccinates** **(*****n*** **= 14)**	PPD B	13; 0.28 (0.06)	13; 0.39 (0.07)	12; 0.26 (0.06)	13; 0.48 (0.08)	13; 0.78 (0.14)
	PPD A	11; 0.28 (0.07)	11; 0.35 (0.09)	8; 0.20 (0.06)	11; 0.44 (0.09)	13; 0.80 (0.17)
	PPD (B-A)	2; 0.01 (0.04)	3; 0.04 (0.04)	5; 0.06 (0.03)	5; 0.05 (0.06)	6;−0.02 (0.08)
	DST	0; 0.01 (0)	0; 0 (0)	0; 0 (0)	0; 0 (0)	1; 0.1 (0.1)
**Vaccinates** **(*****n*** **= 12)**	PPD B	9; 0.25 (0.10)	9; 0.16 (0.01)	6; 0.42 (0.24)	11; 0.96 (0.28)	12; 0.96 (0.18)
	PPD A	8; 0.21 (0.06)	8; 0.19 (0.05)	6; 0.33 (0.22)	11; 1.11 (0.32)	12; 1.14 (0.23)
	PPD (B-A)	2; 0.03 (0.10)	0;−0.04 (0.04)	4; 0.09 (0.04)	1; −0.15 (0.10)	0; −0.19 (0.10)
	DST	2; 0.04 (0.03)	0; 0 (0)	0; 0.01 (0)	1; 0.01 (0.01)	1; 0.01 (0.01)

*Mean IGRA responses of background-corrected optical density (OD) values for PPD-A, PPD-B, and DST at each timepoint were calculated. BOVIGAM IGRA interpretation of PPD-B minus PPD-A is also shown. Calves were revaccinated with BCG after 6 months at day 0, where blood was collected for IGRA prior to injection with BCG and at days 15, 30, 45, and 60 post vaccination. Skin test was conducted at day 61 with PPDs and DST*.

### DST Retains DIVA Capability in BCG Revaccinates

**Figure 3:**
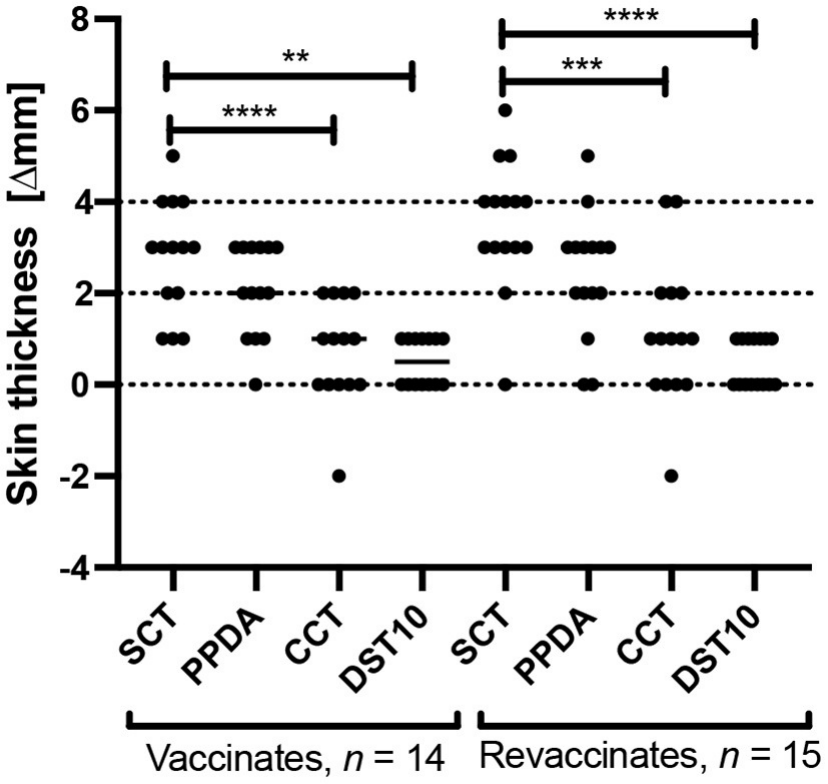
Skin test responses induced by PPD-B (SCT), PPD-A, CCT, and DST10 (10 μg per peptide constituent) were measured 72 h post injection in calves vaccinated with BCG for the first time (*n* = 14) and in revaccinates (*n* = 15). Results are expressed as the difference in skin thickness (in millimeters) between pre- and post-skin test readings, with the horizontal line providing the median [±95% confidence interval (CI)]. The statistical difference between the responses was determined using Tukey's multiple comparison test (****p < 0.0001, ***p < 0.001, **p < 0.01). The dotted horizontal lines at 2 and 4 mm are the cutoffs used for DST, and SCT and CCT, respectively.

A skin test was conducted once at the end of the trial (day 61) on all animals in order to test the specificity of DST post a second dose of BCG. Tuberculin antigens PPD-A and PPD-B were also injected alongside DST. Per SCT (involving PPD-B only; cut-off of ≥4 mm), 8/15 revaccinates and 4/14 vaccinates were found to be reactors (Figure 3). In contrast, the DST induced only minimal responses in both vaccinates and revaccinates, and none of the animals crossed the 2 mm cut-off at 72 h post-injection. The responses induced by SCT was also found to be significantly greater than those of comparative cervical tuberculin (CCT) and DST in both vaccinates and revaccinates (Tukey's multiple comparison test; ^****^p < 0.0001, ^***^p < 0.001, ^**^p < 0.01). Per the CCT, none of the vaccinates and revaccinates were detected as positive (using >4 mm as the criteria) due to equally high PPD-A responses (Figure 3).

## Discussion

In the early 1900s, the studies conducted by Calmette and Guérin demonstrated the incomplete protection conferred by a single dose of BCG vaccination and the need for revaccination (26). However, despite the early establishment of the need for revaccination to help boost immunity, there have been very few studies conducted on the duration of immunity conferred by BCG and optimal revaccination intervals required (27–31). While BCG is the most widely used of all human vaccines, it is not a licensed vaccine for domestic livestock due to the sensitization and interference with the OIE-recommended tuberculin-based skin tests (32). In order to address this critical issue, a new diagnostic test that cannot only reliably detect infection but also differentiate infection and vaccination is needed (33). Antigen-mining strategies and microarray technology have facilitated the development of methods to identify and validate DIVA antigens (34). Several potential candidates were identified, of which some showed promise. Comparative genomic analyses revealed that genes encoding ESAT6 and CFP10, located on the RD1 region of *M. bovis* and *M. tuberculosis*, is deleted in all BCG strains (17, 35–37). It was also shown that the test sensitivity increases when the two antigens are used in combination compared with when either ESAT-6 or CFP-10 was used alone. Most importantly, there was no damage to test specificity when the ESAT-6/CFP-10 cocktail was tested in BCG-vaccinated cattle (38). Similarly, comparative transcriptomic analyses revealed another antigen, Rv3615c, which cannot be secreted by BCG, although it is present in the genome (18). Rv3615c was observed to improve sensitivity of the ESAT-6/CFP-10 cocktail by identifying animals that were not detected by ESAT-6/CFP-10 (39). Recently, Srinivasan et al. developed a peptide-based defined antigen skin test (DST) comprising of three antigens (ESAT-6, CFP-10, and Rv3615c) and showed comparable sensitivity in known reactors and significantly higher specificity in naïve controls, with that of the SCT. Most importantly, specificity in BCG-vaccinated calves was also established in field conditions in India wherein DST was shown to have a perfect specificity of 100% (one-tailed lower 95% CI: 82) (19). In continuation of these studies, we here assess the specificity of DST in revaccinated animals given that a future field roll-out of BCG will likely require administration of more than a single dose of vaccination.

The current study was conducted for a period of 60 days, with two groups of calves viz, vaccinates (*n* = 14) and revaccinates (*n* = 15). The calves were recruited from bTB-free farms and housed in a separate barn that belongs to TANUVAS in Chennai, India. Similar to our previous study, despite BCG's greater genetic similarity with PPD-B than PPD-A, both PPD-A and PPD-B elicited equivalently high responses starting at day 0 and peaked at day 60. We note here that while the trajectories of IGRA responses observed in both the trials were different, they were not statistically significant (Supplementary Figure S1). These differences may be attributed to age at which the different groups were vaccinated, timing of IGRA reflecting length of exposure to environmental mycobacteria, timing of skin test, etc. The BCG-induced cross-reactivity was also observed in skin test wherein both PPDs elicited non-specific responses. Per SCT (PPD-B), four vaccinates and eight revaccinates were detected as reactors, while none of the animals crossed the CCT interpretation of >4 mm due to equally high PPD-A and PPD-B responses. In this context, it is also important to note that in regions where there is high burden of bTB and environmental mycobacteria, there are serious implications for the use of PPD-based tests. In these settings, PPD-A reactivity is driven by non-tuberculous mycobacteria, and therefore, we acknowledge that the OIE-recommended CCT would have a high rate of false negativity, whereas SCT suffers poor specificity (high false positivity). Importantly, none of the animals showed any measurable skin test response to DST, highlighting the high diagnostic specificity of these antigens even post a second dose of BCG.

There were several limitations to the study. First, the sample size is low, and the results are required to be validated in larger trials. Second, we are unable to establish true assay specificity due to the lack of bacteriological and post-mortem data confirming disease-free status. However, given that the animals were recruited from disease-free farms and stayed negative to standard diagnostic tests for the duration of the trial, this may not have a major impact on study findings. Third, duration of the current study is shorter than the previous trial, and hence, the diagnostic specificity of DST in revaccinates is unknown 2 months post-revaccination. Further studies are needed to assess the performance of DST beyond this timepoint.

In light of the predicted intensification of dairy farming in settings such as India to meet increased economic and nutrition demands, the current burden of bTB will likely worsen in the near future. Hence, there is an urgent need to implement practical and affordable solutions for the control of bTB. Herd-level transmission dynamic models and scenario analyses have recently highlighted that despite BCG being a leaky vaccine, it may be good enough to help control bTB, particularly if implemented sooner rather than later (13). Furthermore, revaccination will be essential given that immunity conferred by BCG has been shown to wane after a 12- to 18-month period. In this scenario, there is an urgent need for a diagnostic test that will retain DIVA capability post-multiple doses of BCG. Here, we confirm that the peptide-based DST retains its high specificity post-revaccination, performed at a more stringent 6-month interval. Large-scale field trials are required to further validate these results and to utilize DST alongside BCG in a national control program.

## Data Availability Statement

The original contributions presented in the study are included in the article/Supplementary Material, further inquiries can be directed to the corresponding author/s.

## Ethics Statement

The animal study was reviewed and approved by Institutional Animal Ethics Committee (IAEC) and Committee for the Purpose of Control and Supervision of Experimental Animals (CPCSEA; F. No. 25/31/2017-CPCSEA), Tamil Nadu University of Veterinary and Animal Sciences (TANUVAS), Chennai, India.

## Author Contributions

SSr, DG, DB, MV, and VK conceptualized and designed the study. SSu, BR, and PV performed the experiments. KR, SSe, and KK carried out the field work. SSr analyzed the data. SSu and SSr drafted the paper. DB, MV, and VK contributed to writing. All authors reviewed the manuscript. All authors contributed to the article and approved the submitted version.

## Funding

This research project has been implemented with financial contributions from the Department of Biotechnology, Government of India under the DBT Network Program on Bovine Tuberculosis Control: Mycobacterial Diseases in Animals Network (MyDAN) Program (Scheme Code No. 22270) and Accelerating Bovine Tuberculosis Control in Developing Countries—India (AbTBCD) program funded by the Bill and Melinda Gates Foundation (Scheme Code No. 27031/OPP1176950). The publication does not constitute an endorsement by the Government of India.

## Conflict of Interest

APHA (MV) and Penn State (SSr and VK) have filed a patent application for DST (U.S. Patent Application 17/602,628). The remaining authors declare that the research was conducted in the absence of any commercial or financial relationships that could be construed as a potential conflict of interest.

## Publisher's Note

All claims expressed in this article are solely those of the authors and do not necessarily represent those of their affiliated organizations, or those of the publisher, the editors and the reviewers. Any product that may be evaluated in this article, or claim that may be made by its manufacturer, is not guaranteed or endorsed by the publisher.
