# Supplementary figures and images for: Defined Antigen Skin Test for Bovine Tuberculosis Retains Specificity on Revaccination With Bacillus Calmette–Guérin
Source: Front Vet Sci. 2022 Apr 13;9:814227. doi: 10.3389/fvets.2022.814227 (PMC9043861; doi:10.3389/fvets.2022.814227)

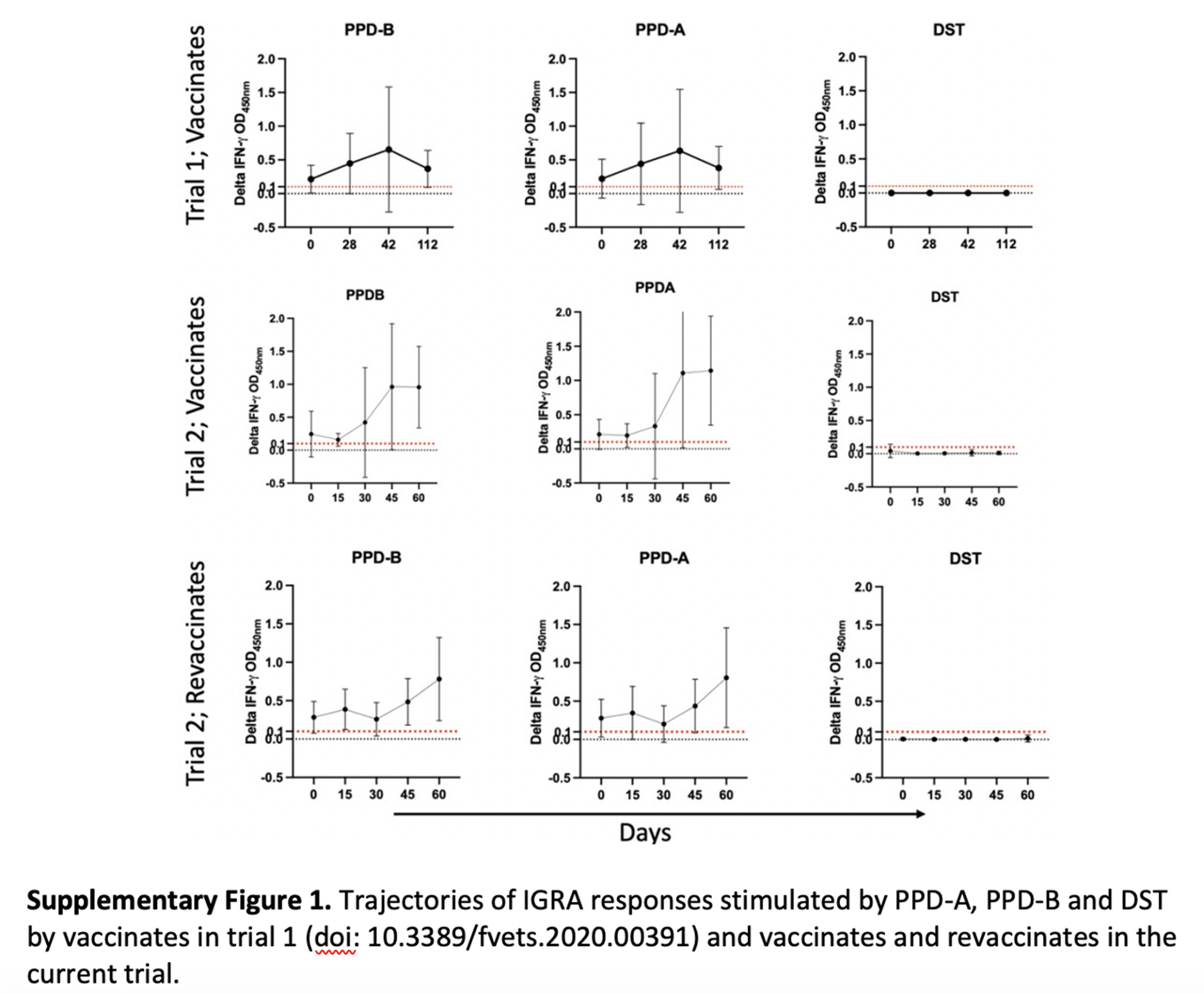

Supplement: Supplementary file 2 [file Image_1.png]
